# Medication and Procedural Abortion and Risk of Psychotropic Medication Use

**DOI:** 10.1001/jamapsychiatry.2025.3698

**Published:** 2026-07-22

**Authors:** Julia R. Steinberg, Thomas M. Laursen, Øjvind Lidegaard, Trine Munk-Olsen

**Affiliations:** 1 Department of Family Science, University of Maryland, College Park; 2 Center for Integrated Register-Based Research, Aarhus University, Aarhus, Denmark; 3 National Centre for Register-Based Research, Aarhus University, Aarhus, Denmark; 4 Department of Clinical Medicine, University of Copenhagen, Copenhagen, Denmark; 5 Research Unit of Child and Adolescent Psychiatry, Department of Clinical Research, University of Southern Denmark, Odense, Denmark

## Abstract

**Importance:**

No recent study has examined whether method of abortion is associated with psychotropic medication use, an indicator of mild mental health problems.

**Objective:**

To examine whether medication or procedural abortions were associated with increased risk of psychotropic medication use.

**Design, Setting, and Participants:**

This population-based Danish registry cohort study followed the psychotropic medication redemptions of females aged 12 to 38 years having elective, first first-trimester abortions between January 1, 2000, and December 31, 2018, from 1 year before their medication or procedural abortion until their first psychotropic medication prescription claim (redemption), December 31, 2018, emigration from Denmark, or death, whichever came first. Analyses were conducted between June 2023 and June 2025.

**Exposure:**

First abortion method (first medication abortion or first procedural abortion).

**Main Outcomes and Measures:**

Any first psychotropic medication prescription redemption, first antidepressant medication redemption, and first antianxiety medication redemption during the study period. Incidence rate ratios (IRRs) were calculated in adjusted regression models. Bonferroni-corrected *P* values were used to account for multiple tests.

**Results:**

Of 67 390 females included in this study (mean [SD] age, 21.8 [4.6] years), 4575 (6.8%) had a prior psychiatric diagnosis; 33 793 had a record of a first medication abortion, 33 597 had a record of a first procedural abortion, and 19 979 (29.6%) had a first psychotropic medication redemption during the study period. In fully adjusted models, using the conventional *P* value level of .05, compared with the year before a medication or procedural abortion, there were small increased risks of any first psychotropic medication redemption in the first year after medication abortion (IRR, 1.10; 95% CI, 1.02-1.19; *P* = .01) and procedural abortion (IRR, 1.09; 95% CI, 1.01-1.16; *P* = .02). There were no statistically significant associations in 1 to 2 years and 2 to 5 years after abortion relative to the year before an abortion for each abortion method. There were decreased risks of any first psychotropic medication redemption more than 5 years after medication abortion (IRR, 0.85; 95% CI, 0.78-0.91; *P* < .001) and more than 5 years after procedural abortion (IRR, 0.83; 95% CI, 0.78-0.88; *P* < .001). Using the Bonferroni-corrected *P* value of .002, the small increased risks of psychotropic medication redemption observed in the first year after an abortion no longer met the criteria for statistical significance, while the lower risks for more than 5 years after abortion continued to meet the criteria for statistical significance.

**Conclusions and Relevance:**

In this Danish population-based cohort study, compared with the year before a medication or procedural abortion, small increased risks of psychotropic medication prescription redemptions were observed during the first year after abortion, and decreases in risk of psychotropic medication prescription redemptions were observed more than 5 years after abortion when using conventional levels of statistical significance. The small increased risks observed in the first year after an abortion no longer met the criteria for statistical significance following Bonferroni adjustment for multiple statistical tests. Additional research may be needed to replicate and understand these results.

## Key Points

**Question:**

Is medication or procedural abortion associated with an increased risk of mild mental health problems as indicated by any first psychotropic medication redemption?

**Findings:**

In this population-based cohort study of 67 390 Danish females who had a first first-trimester abortion between January 1, 2000, and December 31, 2018, compared with the year before a medication abortion or procedural abortion, there were small increased risks of any first psychotropic medication prescription claims (redemptions) in the first year after abortion, no increased risk of psychotropic medication prescription redemptions between 2 to 5 years after abortion, and a decreased risk in more than 5 years after abortion when using the conventional *P* value level of .05. Following Bonferroni adjustment for multiple tests (*P* < .002), compared with the year before a medication abortion or procedural abortion, the risks in the first year after the abortion no longer met criteria for statistical significance, while the lower risks more than 5 years after abortion continued to meet the criteria for statistical significance.

**Meaning:**

The small increased risks for any first psychotropic medication prescription redemptions in the first year after an abortion observed in this study no longer met the criteria for statistical significance following Bonferroni adjustment; additional research may be needed to replicate and understand these results.

## Teaser

This population-based Danish registry cohort study examines whether medication abortions or procedural abortions were associated with increased risk of psychotropic medication use.

## Introduction

The proportion of abortions provided by medication has steadily increased in the US^1^ and worldwide^2,3,4^ since the US Food and Drug Administration approved mifepristone in 2000. It is estimated that 63% of abortions in the US in 2023 and 75% in Denmark in 2018 were provided by medication.^1,4^ Despite the overturning of *Roe v Wade* in June 2022, the number of abortions provided overall and by medication in the US appears to have increased.^5,6,7,8^ The proportion of medication abortions also appears to have increased to approximately 80% in Denmark in more recent years.^9^ While research has shown that medication abortion is as physically safe as procedural (also known as *surgical*) abortion^10,11^ and is not associated with subsequent adverse pregnancy outcomes,^10,12^ there is limited recent research on whether medication or procedural abortion increases the risk of mental health problems.^13^

Our recent study using Danish population registry data found that neither medication nor procedural abortions increased risk of any first psychiatric diagnoses, a first mood disorder diagnosis, or a first anxiety or stress disorder diagnosis.^13^ In Denmark, psychiatric diagnoses by mental health professionals, which the aforementioned study focused on, are usually indicative of more severe mental health problems than those for which psychotropic medications are prescribed by general practitioners.^14,15^ Thus, the current study extends our previous research by examining the outcomes of any prescription claim (ie, redemption) for psychotropic medication, antidepressant medication, and antianxiety medication, which are frequently prescribed by general practitioners and so are usually indicative of less severe mental health problems relative to mental health diagnoses from mental health professionals in Denmark. Moreover, given the recent request that the US Food and Drug Administration review data on the effects of mifepristone^16^ and the claims that medication abortion is harmful to women’s mental health,^17^ more research on mental health around both medication and procedural abortion is warranted.

## Methods

We followed the Strengthening the Reporting of Observational Studies in Epidemiology (STROBE) reporting guidelines to enhance clarity and transparency.^18^ This study was deemed exempt by the institutional review board of the University of Maryland, College Park and was approved by the Danish Protection Agency, the Danish National Board of Health, and Statistics Denmark. All data were deidentified. According to Danish law, informed consent is not required for register-based studies.

### Study Population

We conducted a cohort study using information on 85 050 females born in Denmark between 1980 and 2006 who did not die or emigrate from Denmark before their 12th birthday or study entry and who had a first first-trimester abortion between January 1, 2000, and December 31, 2018. We examined abortions in 2000 and later because medication abortion only became available in Denmark in December 1997.^19^ Follow-up started up to 1 year before females’ first first-trimester abortion and ended at the date of first psychotropic medication retrieval, date of emigrating from Denmark, date of death, or December 31, 2018, whichever came first. Thus, the earliest date follow-up began was January 1, 1999, and the youngest age was 12 years. Because our focus is on first psychotropic medication use, we excluded females who had a recorded psychotropic medication retrieval before age 12 years or before 1999 (n = 1728) or more than 1 year before their first abortion (n = 15 932), leaving a total of 67 390 females for analysis. Analyses were conducted between June 2023 and June 2025.

### Measures

#### Exposures: First Abortion Method (Medication or Procedural)

During the study period, we examined elective medication or procedural abortions up to 11 weeks 6 days (83 days) of gestation for any reason. We examined pre-post changes after medication and procedural abortions in 4 categories: within 1 year after, 1 to 2 years after, 2 to 5 years after, and more than 5 years after abortion. We did not examine time after spontaneous abortions (or miscarriages). The comparison was the year before a medication or procedural abortion. In Denmark at the time of these data, abortions were provided by the public health care system and permitted up to 11 weeks 6 days’ gestation. For individuals seeking abortion, physicians encourage medication abortion up to 8 completed weeks (<56 days after last menstrual period). Most females choose procedural abortion from 9 to 11 completed weeks (63-83 days) of gestation and later. First records of first-trimester abortions (*International Statistical Classification of Diseases and Related Health Problems, Tenth Revision *[*ICD-10*] code O04) were identified through the Danish National Patient Register^20^ and the Danish National Induced Abortion Register.^21^ A total of 2500 females who had records of both medication and procedural abortion at their first first-trimester abortion were defined as procedural. They included those who were receiving misoprostol to soften their uterus before a procedural abortion or who were transferred from having a medication abortion to a procedural abortion because the medication was unsuccessful or because of severe bleeding (O.L. and J.R.S., email communication, January 24, 2022).

#### Outcome: Psychotropic Medication Redemptions as Indicators of Mild to Moderate Mental Disorders

Our main outcome was first psychotropic medication retrieval identified from the Danish National Prescription Register, defined as having any records of a psychotropic medication prescription claim (ie, redemption) from a Danish pharmacy during the study period.^20,22^ The medications we examined included antidepressant medications (Anatomical Therapeutic Chemical [ATC] codes of N06A), antianxiety medications (N05C, N05BA, or N05AE01), antipsychotic medications (N05A), mood stabilizers (N03AG01, N03AX09, N03AF01, or N03AF02), and attention-deficit/hyperactivity disorder medications (N06BA04, C02AC01, C02AC02). Because of claims that medication abortion is psychologically traumatic,^17^ we also separately examined first antidepressant medication redemptions (ATC code N06A) and antianxiety medication redemptions (ATC codes N05C, N05BA, or N05AE01).

### Covariates

We included the time-varying covariate calendar year and the time-invariant covariates of age, gestational age, and partner status at the time of abortion, childhood economic background (mother’s and father’s income when the female was aged 12 years), and father’s presence during childhood (indicated by a link or no link to the female’s father in the Danish registries). We also examined presence of the following covariates before 1 year before the abortion: mother’s and father’s psychiatric diagnoses, any psychiatric diagnoses for the female, childbirth history (yes vs no), and physical health (Charlson Comorbidity Index). These covariates were selected as they have been found to be associated with mode of abortion or mental health.^23,24,25^ Information was drawn from the Danish Civil Registration System,^26^ Danish National Induced Abortion Register,^21^ Danish National Patient Register,^20^ Danish Psychiatric Central Research Register,^22^ and Statistics Denmark: Labour and Income Social Statistics.^27^

### Statistical Analysis

We compared study population characteristics by abortion method using Pearson χ^2^ tests. We compared incidence rates per 1000 female-years and 95% CIs for any first psychotropic medication, antidepressant medication, or antianxiety medication redeemed by covariates using Wald χ^2^ tests. We conducted 2 sets of analyses. First, we analyzed data using a Poisson regression model with person-years at risk as an offset variable, approximating a Cox regression model. In Cox regression, proportional hazards are assumed across the entire follow-up. In Poisson regression, this assumption was only needed within the chosen time intervals.^28^ In 3 separate analyses, we examined the risk of any first psychotropic, first antidepressant, and first antianxiety medication redemption associated with a first medication abortion or a first procedural abortion. For these analyses, we examined incidence rate ratios (IRRs) comparing rates in the time after an abortion (≤1 year after, >1 to 2 years after, >2 to 5 years after, and >5 years after) relative to those in the year before, within each abortion method. We used Wald χ^2^ tests to compare IRRs. We examined an unadjusted model, a basic model that included only the time-varying covariate of calendar year, and a fully adjusted model that adjusted for calendar year and all time-invariant covariates. Because for each model we made 24 comparisons or tests, we applied a Bonferroni correction to the conventional *P* value level of .05.^29,30^ This meant we divided the *P* value by 24 to achieve a Bonferroni-corrected *P* value level of .002 to account for multiple tests. All *P* values were 2-tailed.

We conducted 2 sensitivity analyses. First, we examined risk of first psychotropic medication redemption associated with a first medication abortion and a first procedural abortion for those having abortions from 8 weeks 0 days to 8 weeks 6 days of gestation (56-62 days after last menstrual period). For this gestational week, clinicians in Denmark do not typically encourage a particular abortion method, and so abortion method largely reflects females’ preferences (O.L. and J.R.S., email communication, October 30, 2020, and November 28, 2022). Second, we included the 2500 females who had both medication and procedural abortion codes categorized in the medication abortion group rather than the procedural abortion group (as we did in the main analyses).

Our second analysis set examined incidence rates of first-time psychotropic medication redemption by abortion method. We examined rates in bimonthly periods from the year before to the year after an abortion within each abortion method to reduce random variation due to few females having the outcomes of interest. We also calculated IRRs using the 11th and 12th months before the medication or procedural abortion as the reference category, adjusting for calendar year, allowing us to examine whether the incidence of a psychotropic medication prescription claim was increased at any time in 10 months before to 12 months after abortion using Wald χ^2^ tests. Like the first analysis set, we used a Bonferroni-corrected *P* value for the multiple tests by dividing the conventional *P* value level of .05 by 11, the number of tests within each model, to achieve *P* = .0045.

We conducted supplementary analyses stratified by gestational age at the time of abortion (<8 completed weeks [or <56 days], 8 completed weeks [or 56-62 days], and 9 to 11 completed weeks [or 63-83 days]) because physicians in Denmark encourage medication use up to 8 completed weeks of pregnancy and most females having abortions in Denmark choose procedural from 9 to 11 completed weeks (63-83 days).

## Results

A total of 67 390 females were included in this study. Of these, 33 793 had a record of a first first-trimester medication abortion, 33 597 had a record of a first first-trimester procedural abortion, and 19 979 (29.6%) had a first psychotropic medication redemption during the study period of January 1, 1999, to December 31, 2018. Of these 19 979, 58.2% (n = 11 622) had an antidepressant medication redemption, 26.9% (n = 5370) had an antianxiety medication redemption, and 2.4% (n = 470) had both an antidepressant and antianxiety medication at first psychotropic medication redemption. The remaining 12.6% (n = 2517) of first psychotropic medications were antipsychotic medications, mood stabilizers, or attention-deficit/hyperactivity disorder medications.

Table 1 presents descriptive information of the sample by abortion method. The mean (SD) age of study participants was 21.8 (4.6) years, and 4575 (6.8%) had any psychiatric diagnosis at least 1 year before their abortion. Table 2 presents incidence rates of first psychotropic medication redemptions by all covariates. All psychotropic medication outcomes differed by each covariate, with the exceptions that all medication redemption outcomes did not differ by father’s presence during childhood and that first antidepressant and antianxiety medication redemptions did not differ by partner status.

**Table 1.** Study Population Characteristics by Abortion Method

Characteristic	No. (%)			*P* value
	Total (N = 67 390)	Medication abortion (n = 33 793)	Procedural abortion (n = 33 597)	
**Time-invariant covariates**				
Characteristics of females				
Age at abortion, y				
12-17	13 239 (19.7)	4491 (13.3)	8748 (26.0)	
≥18	54 151 (80.4)	29 302 (86.7)	24 849 (74.0)	
Mean (SD)	21.8 (4.6)	22.8 (4.8)	20.7 (4.0)	<.001
Gestational age at abortion				
<8 wk, or <56 d	38 846 (57.6)	29 673 (87.8)	9173 (27.3)	<.001
8 wk, or 56-62 d	11 726 (17.4)	3452 (10.2)	8274 (24.6)	
9-11 wk, or 63-83 d	16 818 (25.0)	668 (2.0)	16 150 (48.1)	
Partner status at abortion				
Married or cohabitating	16 344 (24.3)	9772 (28.9)	6572 (19.6)	<.001
Neither married nor cohabitating	51 046 (75.7)	24 021 (71.1)	27 025 (80.4)	
Any psychiatric diagnosis				
No	62 815 (93.2)	31 603 (93.5)	31 212 (92.9)	.001
Yes	4575 (6.8)	2190 (6.5)	2385 (7.1)	
Childbirth history^a^				
No	57 005 (84.6)	27 407 (81.1)	29 598 (88.1)	<.001
Yes	10 385 (15.4)	6386 (18.9)	3999 (11.9)	
Charlson Comorbidity Index^a^				
0	62 113 (92.2)	31 077 (92.0)	31 036 (92.4)	<.001
1	4415 (6.6)	2225 (6.6)	2190 (6.5)	
≥2	862 (1.3)	491 (1.5)	371 (1.1)	
Characteristics of females’ parents or childhood environment				
Father’s presence during childhood^b^				
No	710 (1.1)	335 (1.0)	375 (1.1)	.11
Yes	66 680 (99.0)	33 458 (99.0)	33 222 (98.9)	
Father’s income at female’s age 12 y				
Lowest third	23 747 (35.2)	11 199 (33.1)	12 948 (38.5)	<.001
Middle third	12 791 (32.3)	10 989 (32.5)	10 490 (31.2)	
Highest third	18 461 (27.4)	10 016 (29.6)	9400 (28.0)	
Unknown	3391 (5.0)	1589 (4.7)	759 (2.3)	
Mother’s income at female’s age 12 y				
Lowest third	24 054 (35.7)	11 106 (32.9)	12 948 (38.5)	<.001
Middle third	21 623 (32.1)	11 133 (32.9)	10 490 (31.2)	
Highest third	20 212 (30.0)	10 812 (32.0)	9400 (28.0)	
Unknown	1501 (2.2)	742 (2.2)	759 (2.3)	
Any parental psychiatric diagnosis				
Father^a^				
No	59 516 (88.3)	30 032 (88.9)	29 484 (87.8)	<.001
Yes	7164 (10.6)	3426 (10.1)	3738 (11.1)	
Unknown father	710 (1.1)	335 (1.0)	375 (1.1)	
Mother^a^				
No	58 905 (84.6)	29 722 (81.1)	29 183 (88.1)	<.001
Yes	8485 (15.4)	4071 (18.9)	4414 (11.9)	
**Time-variant covariate**				
Calendar year				
2000-2004	10 893 (16.2)	2170 (6.4)	8723 (26.0)	<.001
2005-2009	18 099 (26.9)	6842 (20.2)	11 257 (33.5)	
2010-2014	21 621 (32.1)	12 525 (37.1)	9096 (27.1)	
2015-2018	16 777 (24.9)	12 256 (36.3)	4521 (13.5)	

^a^ Assessed before 1 year before abortion.

^b^ Indicated by whether the female had a link to her father in the Danish registers.

**Table 2.** Incidence Rates per 1000 Female-Years for Any First Psychotropic Medication, Antidepressant Medication, or Antianxiety Medication Redeemed Among 67 390 Females

Characteristic	Any first psychotropic medication		Any first antidepressant		Any first antianxiety medication	
	No./1000 female-years (95% CI)	*P* value	No./1000 female-years (95% CI)	*P* value	No./1000 female-years (95% CI)	*P* value
**Time-invariant covariates**						
Characteristics of females						
Age at abortion, y						
12-17	46.7 (45.4-47.9)	<.001	27.2 (26.2-28.2)	<.001	12.3 (12.0-12.7)	<.001
≥18	40.5 (39.9-41.2)		24.8 (24.3-25.3)		11.9 (11.3-12.6)	
Gestational age at abortion						
<8 wk, or <56 d	39.6 (38.8-40.4)	<.001	23.3 (22.7-23.9)	<.001	12.0 (11.5-12.4)	<.001
8 wk, or 56-62 d	43.3 (42.0-44.6)		26.5 (25.5-27.6)		12.3 (11.6-13.1)	
9-12 wk, or 63-83 d	45.4 (44.2-46.5)		28.3 (27.5-29.3)		12.7 (12.1-13.3)	
Partner status at abortion						
Married or cohabitating	40.1 (38.8-41.3)	.001	25.1 (24.1-26.1)	.92	12.5 (11.9-13.3)	.89
Neither married nor cohabitating	42.4 (41.8-43.1)		25.4 (24.9-25.9)		12.2 (11.8-12.5)	
Any psychiatric diagnosis^a^						
No	39.4 (38.9-40.0)	<.001	53.3 (50.4-56.3)	<.001	11.8 (11.5-12.1)	<.001
Yes	89.7 (86.0-93.6)		23.9 (23.5-24.4)		21.2 (19.4-23.2)	
Childbirth history^a^						
No	40.9 (40.3-41.5)	<.001	24.4 (23.9-24.9)	<.001	12.0 (11.7-12.3)	<.001
Yes	49.9 (48.0-51.8)		32.9 (31.4-34.4)		14.1 (13.2-15.1)	
Charlson Comorbidity Index^a^						
0	41.0 (40.4-41.6)	<.001	24.8 (24.3-25.3)	<.001	12.0 (11.7-12.4)	<.001
1	55.1 (52.4-58.0)		33.3 (31.2-35.6)		15.4 (13.9-16.9)	
≥2	52.3 (46.3-59.1)		33.0 (28.3-38.5)		13.2 (10.3-16.8)	
Characteristics of females’ parents or childhood environment						
Father’s presence during childhood^b^						
No	45.7 (40.1-52.0)	.20	24.5 (20.6-29.3)	.71	15.0 (11.9-18.8)	.09
Yes	41.9 (41.3-42.5)		25.4 (24.9-25.8)		12.2 (11.9-12.5)	
Father’s income at female’s age 12 y						
Lowest third	47.5 (46.5-48.6)	<.001	28.4 (27.6-29.3)	<.001	13.6 (13.03-14.2)	<.001
Middle third	42.1 (41.1-43.2)		26.0 (25.2-26.8)		11.7 (11.18-12.3)	
Highest third	34.5 (33.5-35.5)		20.6 (19.9-21.4)		11.1 (10.54-11.7)	
Unknown	44.1 (41.5-46.8)		26.5 (24.5-28.6)		12.7 (11.31-14.2)	
Mother’s income at female’s age 12 y						
Lowest third	50.4 (49.3-51.5)	<.001	30.6 (29.7-31.4)	<.001	14.2 (13.6-14.7)	<.001
Middle third	40.2 (39.2-41.3)		24.7 (23.9-25.5)		11.7 (11.1-12.2)	
Highest third	33.9 (33.0-34.9)		20.0 (19.3-20.8)		10.6 (10.1-11.1)	
Unknown	45.7 (41.7-50.0)		27.7 (24.6-31.1)		13.4 (11.3-15.8)	
Any parental psychiatric diagnosis						
Father^a^						
No	40.2 (39.6-40.8)	<.001	24.4 (23.9-24.8)	<.001	11.8 (11.5-12.1)	<.001
Yes	57.9 (55.8-60.2)		34.7 (33.0-36.4)		16.3 (15.1-17.5)	
Unknown father	45.7 (40.1-52.0)		24.5 (20.6-29.3)		15.0 (11.9-18.8)	
Mother^a^						
No	39.8 (39.2-40.4)	<.001	24.2 (23.7-24.7)	<.001	11.6 (11.3-12.0)	<.001
Yes	59.2 (57.2-61.4)		34.7 (33.1-36.3)		17.0 (16.0-18.2)	
**Time-variant covariate**						
Calendar year						
2000-2004	42.1 (40.0-44.3)	<.001	24.2 (22.6-25.9)	<.001	15.1 (13.8-16.4)	<.001
2005-2009	54.3 (52.9-55.8)		36.5 (35.3-37.7)		14.6 (13.9-15.4)	
2010-2014	46.4 (45.4-47.5)		30.2 (29.7-31.0)		11.4 (10.9-11.9)	
2015-2018	31.1 (30.3-31.9)		15.1 (14.6-15.7)		11.2 (10.7-11.7)	

^a^ Assessed before 1 year before abortion.

^b^ Indicated by whether the female had a link to her father in the Danish registers.

### Comparing Risk in Time After Medication and Procedural Abortions Relative to 1 Year Before

Table 3 shows the crude incidence rates of first psychotropic medication redemption, first antidepressant medication redemption, and first antianxiety medication redemption in the year before, 1 year after, 1 to 2 years after, 2 to 5 years after, and more than 5 years after a medication or procedural abortion. Table 3 also shows the risk of first psychotropic medication redemption, first antidepressant medication redemption, and first antianxiety medication redemption relative to the year before an abortion for those having medication and procedural abortions. Unadjusted models, models adjusted for only calendar year (basic model), and models adjusted for all covariates (fully adjusted model) are presented. We also present findings by the Bonferroni-corrected *P* value of .002.

**Table 3.** Incidence Rates and Incidence Rate Ratios (IRRs) of First Psychotropic Medication Prescription by Time Since Abortion Among 67 390 Females Having a First First-Trimester Medication or Procedural Abortion^a^

Timing	Crude incidence rate, No./1000 female-years (95% CI)	Model 1: unadjusted		Model 2: basic model^b^		Model 3: fully adjusted model^c^	
		IRR (95% CI)	*P* value	IRR (95% CI)	*P* value	IRR (95% CI)	*P* value
**Outcome: any first psychotropic medication prescription**							
Medication abortion							
1 y Before	40.5 (38.4-42.8)	1 [Reference]	NA	1 [Reference]	NA	1 [Reference]	NA
1 y After	43.6 (41.3-46.0)	1.08 (0.996-1.16)	.06	1.10 (1.02-1.18)	.02^d^	1.10 (1.02-1.19)	.01^d^
>1-2 y After	41.4 (38.9-43.9)	1.02 (0.94-1.11)	.62	1.05 (0.97-1.14)	.21	1.06 (0.97-1.15)	.18
>2-5 y After	37.4 (35.9-39.0)	0.92 (0.86-0.99)	.03^d^	0.98 (0.91-1.05)	.55	0.98 (0.92-1.05)	.63
>5 y After	29.8 (28.5-31.3)	0.74 (0.69-0.79)	<.001^e^	0.85 (0.79-0.91)	<.001^e^	0.85 (0.78-0.91)	<.001^e^
Procedural abortion							
1 y Before	49.9 (47.5-52.4)	1 [Reference]	NA	1 [Reference]	NA	1.00	NA
1 y After	54.9 (52.3-57.6)	1.10 (1.03-1.18)	.006^d^	1.08 (1.01-1.16)	.03^d^	1.09 (1.01-1.16)	.02^d^
>1-2 y After	50.8 (48.2-53.5)	1.02 (0.95-1.09)	.63	0.99 (0.92-1.06)	.72	1.00 (0.93-1.07)	.90
>2-5 y After	48.8 (47.2-50.5)	0.98 (0.92-1.04)	.47	0.95 (0.89-1.01)	.09	0.96 (0.90-1.02)	.19
>5 y After	38.1 (37.0-39.3)	0.76 (0.72-0.81)	<.001^e^	0.83 (0.78-0.88)	<.001^e^	0.83 (0.78-0.88)	<.001^e^
**Outcome: any antidepressant prescription**							
Medication abortion							
1 y Before	25.1 (23.4-26.9)	1 [Reference]	NA	1 [Reference]	NA	1 [Reference]	NA
1 y After	26.1 (24.3-28.0)	1.04 (0.94-1.15)	.46	1.07 (0.97-1.18)	.15	1.08 (0.98-1.19)	.14
>1-2 y After	24.9 (23.0-26.9)	0.99 (0.89-1.10)	.87	1.05 (0.94-1.16)	.40	1.05 (0.95-1.16)	.36
>2-5 y After	21.4 (20.3-22.7)	0.85 (0.78-0.93)	<.001^e^	0.94 (0.86-1.03)	.21	0.95 (0.87-1.04)	.24
>5 y After	16.4 (15.4-17.5)	0.65 (0.60-0.72)	<.001^e^	0.84 (0.76-0.93)	<.001^e^	0.83 (0.76-0.92)	<.001^e^
Procedural abortion							
1 y Before	29.6 (27.8-31.6)	1 [Reference]	NA	1 [Reference]	NA	1 [Reference]	NA
1 y After	33.6 (31.6-35.7)	1.13 (1.04-1.24)	.006^d^	1.11 (1.01-1.21)	.03^d^	1.11 (1.02-1.22)	.02^d^
>1-2 y After	31.7 (29.7-33.9)	1.07 (0.98-1.17)	.14	1.03 (0.94-1.13)	.52	1.04 (0.95-1.14)	.39
>2-5 y After	31.2 (29.9-32.5)	1.05 (0.98-1.14)	.18	1.02 (0.94-1.10)	.62	1.04 (0.96-1.12)	.38
>5 y After	23.1 (22.2-24.0)	0.78 (0.72-0.84)	<.001^e^	0.91 (0.84-0.98)	.02^d^	0.92 (0.85-0.99)	.03^d^
**Outcome: any antianxiety prescription**							
Medication abortion							
1 y Before	10.2 (9.2-11.4)	1 [Reference]	NA	1 [Reference]	NA	1 [Reference]	NA
1 y After	12.4 (11.2-13.8)	1.21 (1.05-1.41)	.01^d^	1.23 (1.06-1.43)	.006^d^	1.25 (1.07-1.44)	.004^d^
>1-2 y After	11.1 (9.9-12.5)	1.08 (0.93-1.27)	.31	1.11 (0.95-1.30)	.20	1.13 (0.97-1.33)	.12
>2-5 y After	11.9 (11.0-12.8)	1.16 (1.02-1.32)	.02^d^	1.21 (1.06-1.38)	.004^d^	1.26 (1.11-1.44)	<.001^e^
>5 y After	10.3 (9.5-11.1)	1.00 (0.88-1.15)	.97	1.09 (0.95-1.24)	.24	1.18 (1.03-1.36)	.02^d^
Procedural abortion							
1 y Before	13.8 (12.6-15.2)	1 [Reference]	NA	1 [Reference]	NA	1 [Reference]	NA
1 y After	15.5 (14.2-17.0)	1.12 (0.99-1.28)	.08	1.13 (0.99-1.29)	.06	1.15 (1.01-1.31)^d^	.04^d^
>1-2 y After	13.2 (11.9-14.6)	0.96 (0.83-1.10)	.51	0.97 (0.84-1.11)	.64	0.99 (0.86-1.14)	.89
>2-5 y After	13.2 (12.4-14.1)	0.96 (0.85-1.07)	.44	0.99 (0.88-1.12)	.90	1.04 (0.92-1.16)	.56
>5 y After	12.0 (11.4-12.6)	0.87 (0.78-0.97)	.009^d^	0.99 (0.88-1.11)	.81	1.07 (0.95-1.21)	.25

Abbreviation: NA, not applicable.

^a^ For each time period after the abortion, the reference is the year before the abortion within the method. There were no significant differences between 1 year before and 1 year after or between 1 year before and more than 1 to 2 years after a medication or procedural abortion in any first psychotropic medication redemption, any first antidepressant redemption, and any first antianxiety medication redemption when using the Bonferroni-corrected *P* value of .002.

^b^ Model includes abortion method (medication and procedural abortion) and cohort (calendar year).

^c^ Model includes abortion method (medication and procedural abortion; cohort (calendar year); age, gestational age, and partner status at the time of abortion; psychiatric diagnosis, childbirth history, Charlson Comorbidity Index, and mother’s and father’s psychotropic drug use before 1 year before abortion; father’s presence during childhood; and mother’s and father’s income at the female’s age of 12 years.

^d^ *P* < .05.

^e^ *P* < .002 (Bonferroni-corrected *P* value).

In the fully adjusted models and using the conventional *P* value level of .05, relative to the year before a medication abortion, there was an increased risk of any first psychotropic medication redemption (IRR, 1.10; 95% CI, 1.02-1.19; *P* = .01) and any first antianxiety medication redemption (IRR, 1.25; 95% CI, 1.07-1.44; *P* = .004) in the year after, as well as any first antianxiety medication redemption in the 2 to 5 years after (IRR, 1.26; 95% CI, 1.11-1.44; *P* < .001) and more than 5 years after (IRR, 1.18; 95% CI, 1.03-1.36; *P* = .02). Relative to the year before a procedural abortion, there was an increased risk of any first psychotropic medication redemption (IRR, 1.09; 95% CI, 1.01-1.16; *P* = .02), any first antidepressant medication redemption (IRR, 1.11; 95% CI, 1.02-1.22; *P* = .02), and any first antianxiety medication redemption (IRR, 1.15; 95% CI, 1.01-1.31; *P* = .04) in the year afterward.

In the fully adjusted models and using the conventional *P* value level of .05, relative to the year before a medication or procedural abortion, there was a decreased risk of any first psychotropic medication redemption more than 5 years afterward (medication abortion: IRR, 0.85; 95% CI, 0.78-0.91; *P* < .001; procedural abortion: IRR, 0.83; 95% CI, 0.78-0.88; *P* < .001) and of any first antidepressant medication redemption more than 5 years afterward (medication abortion: IRR, 0.83; 95% CI, 0.76-0.92; *P* < .001; procedural abortion: IRR, 0.92; 95% CI, 0.85-0.99; *P* = .03).

When using the Bonferroni-adjusted *P* value of .002 that accounts for multiple tests for each model, relative to the year before a medication or procedural abortion, the small increased risk of any first psychotropic medication redemption no longer met the criteria for statistical significance, with *P* values of .01 and .02, respectively. However, an increased risk of any first antianxiety medication redemption 2 to 5 years after a medication abortion continued to meet criteria for statistical significance (*P* < .001). In addition, there was a decreased risk of any first psychotropic medication redemption more than 5 years after a medication or procedural abortion and a decreased risk of any first antidepressant medication redemption more than 5 years after a medication abortion.

For the supplementary analysis involving only females having abortions from 8 weeks 0 days to 8 weeks 6 days of gestation (56-62 days), when physicians in Denmark do not encourage a particular abortion method, there were no elevated risks of any first psychotropic, antidepressant, or antianxiety medication redemptions in any model after a medication or procedural abortion relative to the year before (eTable 1 in Supplement 1). The other supplementary analyses presented for 2500 women with both medication and procedural *ICD-10* codes that were placed in the medication abortion group, relative to the year before a medication or procedural abortion, showed increased risks for any psychotropic medication redemption in the year after abortion and a decreased risk for any psychotropic medication redemption more than 5 years after abortion using the conventional *P* value level of .05. There was also an increased risk in antidepressant medication redemption in the year after a procedural abortion relative to the year before an abortion and an increased risk in antianxiety medication redemption in the year after a medication abortion relative to the year before an abortion. When we used the Bonferroni-adjusted *P* value of .002, the only risk that remained statistically significant was that for the 1 year after a medication abortion for antianxiety medication redemptions. The IRRs, 95% CIs, and *P* values for these and other findings are shown in eTable 2 in Supplement 1.

### Rates and Risk of Psychotropic Medication Redemption Around Medication and Procedural Abortions

**Figure 1.**
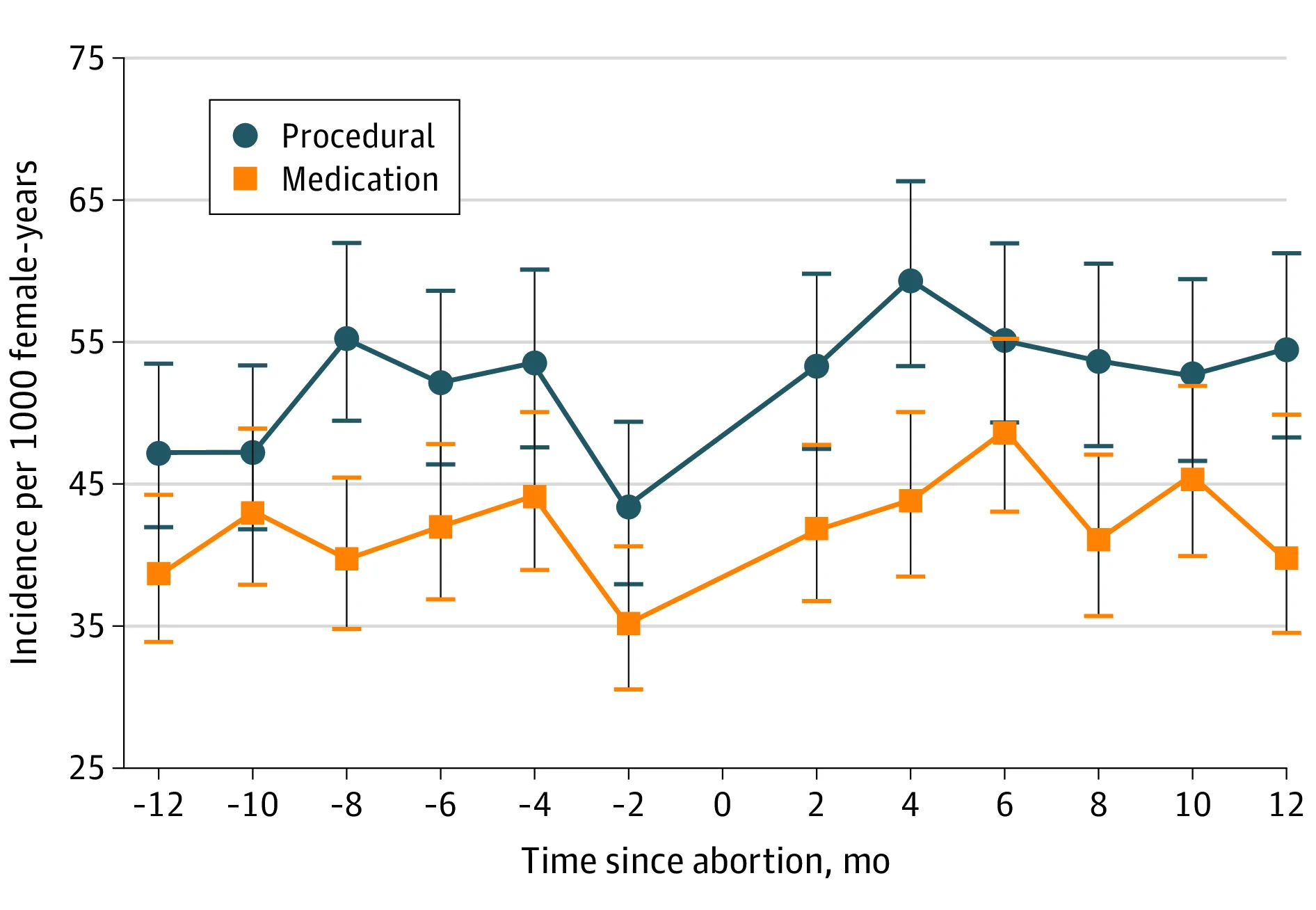
Line Graph of the Incidence Rate of Any Psychotropic Medication Use in the Year Before and Year After Abortion by Abortion Method

**Figure 2.**
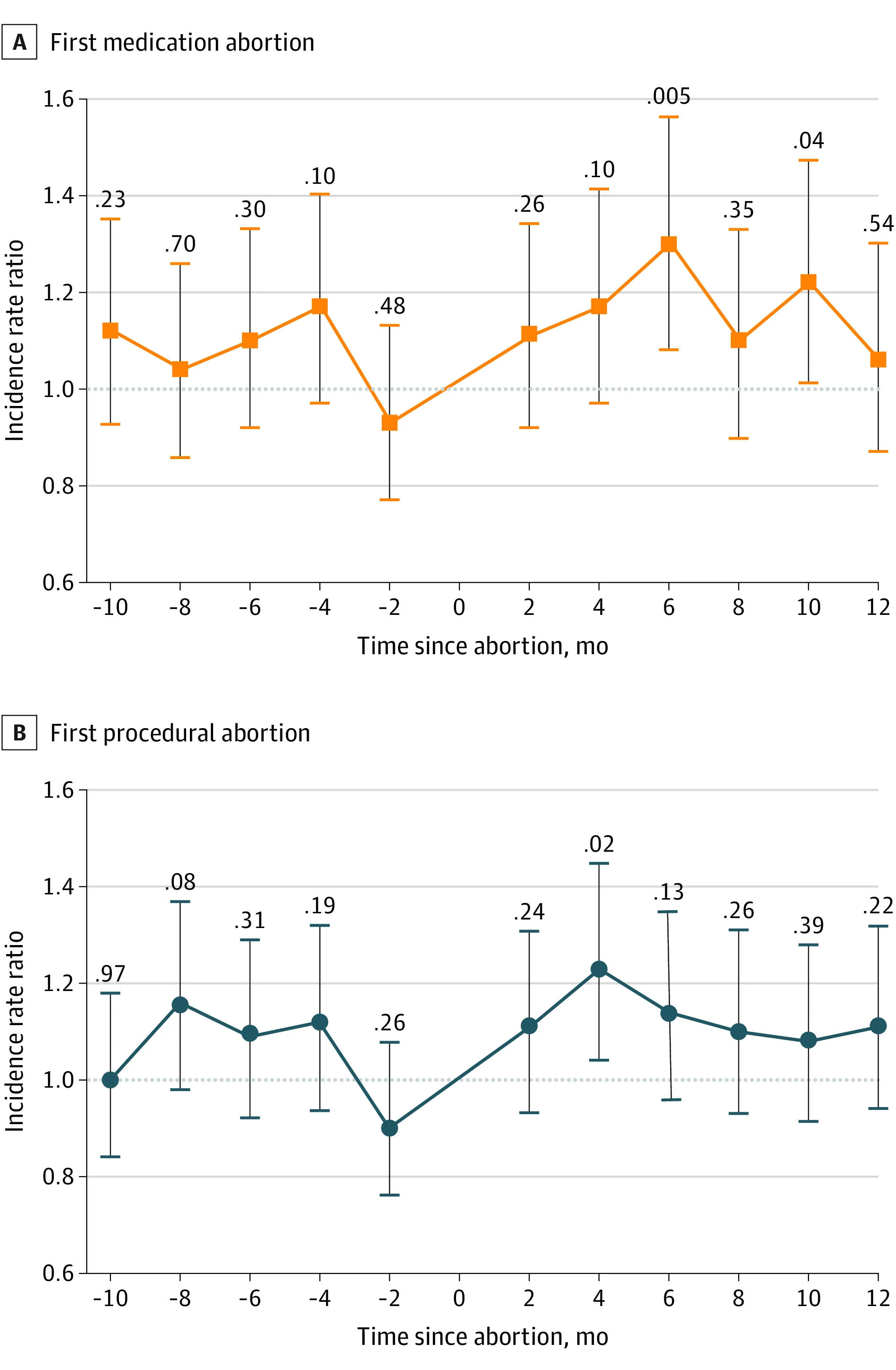
Line Graphs of Incidence Rate Ratios of Any Psychotropic Medication Use in the Year Before and Year After an Abortion, Adjusted for Calendar Year Incidence rate ratios of any psychotropic medication use in the year before and year after an abortion are shown for females having had a first medication abortion (A) and a first procedural abortion (B), adjusted for calendar year. The period of −10 to 0 months refers to the period of 10 months before the abortion. Dotted lines indicate the incidence rate ratio reference of 1.00, which is the incidence rate ratio over the 11th and 12th months before the abortion method. Within each abortion method, there were no consistently higher rates in the 10 months before to the 12 months after abortion relative to the 11th to 12th month before an abortion. Error bars indicate 95% CI. *P* values listed above data points indicate differences between the respective time point and the 11th and 12th month before an abortion.

Figure 1 depicts crude bimonthly incidence rates of any first psychotropic medication redemption in the year before and year after an abortion for each abortion method (eFigure 1 in Supplement 1 shows findings for each abortion method by gestational age at abortion). Figure 2 depicts the calendar year–adjusted IRRs comparing each bimonthly period in the 10 months before to the 12 months after relative to the 11th and 12th months before an abortion for each abortion method. As can be seen in Figure 2, relative to the 11th and 12th months before a medication abortion, there were higher rates of any first psychotropic medication redemptions in the 5th and 6th months (IRR, 1.30; 95% CI, 1.08-1.56; *P* = .005) and 9th and 10th months (IRR, 1.22; 95% CI, 1.01-1.47; *P* = .04) after a medication abortion and the 3rd and 4th months after a procedural abortion (IRR, 1.23; 95% CI, 1.04-1.45; *P* = .02). None of these differences met criteria for statistical significance at the Bonferroni-corrected *P* value of .0045. eFigure 2 in Supplement 1 shows IRRs for any psychotropic medication use in the year before and year after abortion, adjusted for calendar year for females having had a first procedural abortion or first medication abortion by gestational age at time of abortion (compared with IRRs at the 11th and 12th months before the abortion). In this analysis, there were no increased risks for any psychotropic medication redemptions.

## Discussion

The current study examined whether medication or procedural abortion was associated with having a first psychotropic medication redemption—any, antidepressant, or antianxiety medications—in the year after an abortion and as more time from the abortion elapsed relative to the year before an abortion. In fully adjusted models, when using the conventional *P* value level of .05, we found small increased risks for any first psychotropic medication redemption and any first antianxiety medication redemption in the year after a first medication abortion and first procedural abortion, relative to the year before. In addition, we found small increased risks in first antidepressant medication redemption in the year after a first procedural abortion and in first antianxiety medication prescriptions in the 2 to 5 years after a first medication abortion. When correcting for multiple tests, no risks of any psychotropic medication redemption within the first 2 years after a medication or procedural abortion relative to the year before met criteria for statistical significance. However, decreased risks of any first psychotropic medication redemption more than 5 years after a medication or procedural abortion relative to the year before and of any first antidepressant medication more than 5 years after a medication abortion continued to meet criteria for statistical significance.

When using the conventional *P* value level of .05, which does not correct for multiple tests, 16 (4 unadjusted, 5 basic-adjusted, and 7 fully adjusted risk ratios) of 72 IRRs indicated that relative to the year before a medication or procedural abortion, there was a statistically significantly increased risk of psychotropic medication redemption after an abortion. When using the Bonferroni-corrected *P* value of .002, only 1 test (a fully adjusted risk ratio) indicated that there was a statistically significantly increased risk of first-time antianxiety medication redemption in the 2 to 5 years after a medication abortion. However, supplementary analyses of females with abortions from 8 weeks 0 days to 8 weeks 6 days of gestation (eTable 1 in Supplement 1), when females are most likely able to choose their abortion method, showed that compared with the year before an abortion, there was no increased risk of any first psychotropic, antidepressant, or antianxiety medication redemption after a medication or procedural abortion at the conventional *P* value level of .05.

Fifteen (7 unadjusted, 4 basic-adjusted, and 4 fully adjusted) risk ratios indicated at the conventional *P* value level of .05 that relative to the year before an abortion, there was a decreased risk of psychotropic medication use after an abortion. When using the Bonferroni-corrected *P* value, 11 (5 unadjusted, 3 basic-adjusted, and 3 fully adjusted) risk ratios indicated that relative to the year before an abortion, there was a decreased risk of psychotropic medication use after an abortion.

We also compared bimonthly incidence rates in the year before and the year after an abortion relative to the 11th and 12th months before an abortion within the 2 abortion methods, adjusting for calendar year. When using the conventional *P* value level of .05, there were small increases in risk of a first psychotropic medication redemption in the 5th and 6th months and 9th and 10th months after a medication and 3rd and 4th months after a procedural abortion. When we corrected for multiple tests using the Bonferroni-corrected *P* value, we found no increased risk of a first psychotropic medication redemption for either medication or procedural abortion for all gestational ages together (Figure 2) and by different gestational ages of pregnancy (eFigure 2 in Supplement 1).

Our results are important because more females globally are using medications to have abortions,^1,2,3,4,8^ and the US Secretary of Health and Human Services has asked the US Food and Drug Administration to review the latest data on mifepristone, which is used for abortion in the US.^16^ Moreover, unsubstantiated claims that medication abortion in particular is harmful to females’ mental health are increasingly being used to restrict access to medication abortion.^17,31,32,33^

Evidence from the current study may not support these claims. First, while we found small increases (at the conventional *P* value level of .05) in risk of some psychotropic medication redemptions after a first medication or procedural abortion relative to the year before, all increases in risk except 1 (antianxiety prescription redemption 2-5 years after medication abortion) were only in the year after and not more than a year after the abortion. We speculate that the reason for these small increases in risk may be due to the following. To receive a prescription for psychotropic medications (our outcome) around an abortion (before or after) in Denmark, women usually visit their general practitioner. To receive an abortion in Denmark, women may go directly to an obstetrician/gynecologist. While symptoms of anxiety or depression (or another mental health issue) may have predated the women’s abortion, these cases are not detected in our data until after their abortion when women may be more likely to seek and redeem their psychotropic medication prescription. Second, the increased risks in the year after did not meet criteria for statistical significance when using the Bonferroni-corrected *P* value. Third, when examining those who had abortions at 8 completed weeks (56-62 days) of pregnancy, when obstetrician/gynecologists in Denmark do not encourage a particular abortion method, there were no increased risks in the year after abortion at the conventional *P* value level.

### Limitations

There are some limitations worth mentioning. First, the data were from the Danish population registers and might not be generalizable to populations of other countries. However, the claim that medication abortion harms females’ mental health is universal for all contexts. Second, we did not directly assess mental health severity and so do not know the severity of mental illness for those receiving psychotropic medications. We examined the outcomes of any first psychotropic medication, antidepressant, and antianxiety medication redemption because in Denmark these are frequently prescribed by general practitioners and are usually indicative of milder mental health disorders.^14,15^ Third, it is possible that some of the antidepressant or antianxiety medications were redeemed for reasons other than depression or anxiety, and we did not measure actual medication use because measures of redeemed prescriptions do not indicate if the women used them. This potential limitation, however, is not specific for the current study, but a general challenge in pharmacoepidemiological studies. Fourth, in large datasets like the Danish population registries, a small increase or decrease that is significant at *P* < .05 (like we found in this study) should be interpreted with caution (particularly when conducting several tests), simply because they tend to become statistically significant in such large datasets. Fifth, the Bonferroni correction controls for false-positive rates (ie, type I errors) and does not address the possibility of false-negative results (ie, type II errors).

## Conclusions

This population-based Danish registry cohort study found small increased risks for psychotropic medication redemptions in the year after a medication or procedural abortion and decreased risks for psychotropic medication redemptions in the 5 or more years after a medication or procedural abortion. The small increases observed in the first year after an abortion no longer met criteria for statistical significance after correction for multiple tests. Additional research may be needed to replicate and understand these results.

## Notes

Supplement 1. **eTable 1.** Incidence Rate Ratios (and 95% CI) of First Psychotropic Medication Prescription, Antidepressant Prescription, and Antianxiety Prescription Comparing Each Time After the Abortion to 1 Year Beforehand Within Method Type for Those Who Had Abortions Between 8 Weeks 0 Days (56 Days) and 8 Weeks 6 Days (62 Days) Gestation (n = 11,726) **eTable 2.** Incidence Rate Ratios (and 95% CI) of First Psychotropic Medication Prescription, Antidepressant Prescription, and Antianxiety Prescription at Each Time Relative to 1 Year Before the Abortion (n = 67,390) Where 2,500 Women With Both Medication and Procedural Codes Are Placed in the Medication Abortion Group **eFigure 1.** Incidence Rate of Any Psychotropic Medication Use in Year Before and Year After Abortion (a) for First Procedural Abortion by Gestational Age at Time of Abortion, and (b) for First Medication Abortion by Gestational Age at Time of Abortion **eFigure 2.** Incidence Rate Ratios of Any Psychotropic Medication Use in Year Before and Year After Abortion, Adjusted for Calendar Year for Females Having Had a (a) First Procedural Abortion by Gestational Age at Time of Abortion, and (b) First Medication Abortion by Gestational Age at Time of Abortion

Supplement 2. Data Sharing Statement
