# Supplementary material for: Medication and Procedural Abortion and Risk of Psychotropic Medication Use
Source: JAMA Psychiatry. 2026 Jul 22:e253698. Online ahead of print. doi: 10.1001/jamapsychiatry.2025.3698 (PMC13392831; doi:10.1001/jamapsychiatry.2025.3698)
Supplement: Supplement 1. — eTable 1. Incidence Rate Ratios (and 95% CI) of First Psychotropic Medication Prescription, Antidepressant Prescription, and Antianxiety Prescription Comparing Each Time After the Abortion to 1 Year Beforehand Within Method Type for Those Who Had Abortions Between 8 Weeks 0 Days (56 Days) and 8 Weeks 6 Days (62 Days) Gestation (n = 11,726) eTable 2. Incidence Rate Ratios (and 95% CI) of First Psychotropic Medication Prescription, Antidepressant Prescription, and Antianxiety Prescription at Each Time Relative to 1 Year Before the Abortion (n = 67,390) Where 2,500 Women With Both Medication and Procedural Codes Are Placed in the Medication Abortion Group eFigure 1. Incidence Rate of Any Psychotropic Medication Use in Year Before and Year After Abortion (a) for First Procedural Abortion by Gestational Age at Time of Abortion, and (b) for First Medication Abortion by Gestational Age at Time of Abortion eFigure 2. Incidence Rate Ratios of Any Psychotropic Medication Use in Year Before and Year After Abortion, Adjusted for Calendar Year for Females Having Had a (a) First Procedural Abortion by Gestational Age at Time of Abortion, and (b) First Medication Abortion by Gestational Age at Time of Abortion [file jamapsychiatry-e253698-s001.pdf]

Supplemental Online Content

Steinberg JR, Laursen TM, Lidegaard Ø, Munk-Olsen T. Medication and procedural abortion and risk of psychotropic medication use. *JAMA Psychiatry*. Published online July 22, 2026. doi:10.1001/jamapsychiatry.2025.3698

eTable 1. Incidence Rate Ratios (and 95% CI) of First Psychotropic Medication Prescription, Antidepressant Prescription, and Antianxiety Prescription Comparing Each Time After the Abortion to 1 Year Beforehand Within Method Type for Those Who Had Abortions Between 8 Weeks 0 Days (56 Days) and 8 Weeks 6 Days (62 Days) Gestation (n = 11,726)
eTable 2. Incidence Rate Ratios (and 95% CI) of First Psychotropic Medication Prescription, Antidepressant Prescription, and Antianxiety Prescription at Each Time Relative to 1 Year Before the Abortion (n = 67,390) Where 2,500 Women With Both Medication and Procedural Codes Are Placed in the Medication Abortion Group
eFigure 1. Incidence Rate of Any Psychotropic Medication Use in Year Before and Year After Abortion (a) for First Procedural Abortion by Gestational Age at Time of Abortion, and (b) for First Medication Abortion by Gestational Age at Time of Abortion
eFigure 2. Incidence Rate Ratios of Any Psychotropic Medication Use in Year Before and Year After Abortion, Adjusted for Calendar Year for Females Having Had a (a) First Procedural Abortion by Gestational Age at Time of Abortion, and (b) First Medication Abortion by Gestational Age at Time of Abortion

This supplemental material has been provided by the authors to give readers additional information about their work.

© 2026 Steinberg JR et al. *JAMA Psychiatry*.

eTable 1. Incidence Rate Ratios (and 95% CI) of First Psychotropic Medication Prescription, Antidepressant Prescription, and Antianxiety Prescription Comparing Each Time After the Abortion to 1 Year Beforehand Within Method Type for Those Who Had Abortions Between 8 Weeks 0 Days (56 Days) and 8 Weeks 6 Days (62 Days) Gestation (n = 11,726)[a]

	Unadjusted	Basic Model[b]	Fully adjusted model[c]
**Outcome: Any First Psychotropic Medication Prescription**			
**Medication abortion**			
1 year before	1.00 (reference)	1.00 (reference)	1.00 (reference)
<1 year after	0.99 (0.78-1.25)	0.99 (0.78-1.26)	0.99 (0.78-1.26)
1-<2 years after	1.03 (0.80-1.31)	1.03 (0.81-1.32)	1.04 (0.81-1.32)
2-<5 years after	0.90 (0.73-1.10)	0.92 (0.74-1.13)	0.92 (0.75-1.14)
5+ years after	0.74* (0.60-0.91)	0.85 (0.69-1.05)	0.87 (0.70-1.08)
**Procedural abortion**			
1 year before	1.00 (reference)	1.00 (reference)	1.00 (reference)
<1 year after	1.09 (0.95-1.25)	1.07 (0.93-1.22)	1.07 (0.93-1.23)
1-<2 years after	0.95 (0.82-1.10)	0.92 (0.79-1.06)	0.93 (0.80-1.07)
2-<5 years after	0.99 (0.88-1.12)	0.95 (0.84-1.07)	0.96 (0.85-1.08)
5+ years after	0.73** (0.65-0.82)	0.77** (0.69-0.88)	0.78** (0.69-0.88)
**Outcome: First Antidepressant Prescription**			
**Medication abortion**			
1 year before	1.00 (reference)	1.00 (reference)	1.00 (reference)
<1 year after	1.07 (0.79-1.44)	1.08 (0.80-1.46)	1.08 (0.80-1.47)
1-<2 years after	1.12 (0.82-1.52)	1.13 (0.83-1.54)	1.14 (0.84-1.55)
2-<5 years after	0.95 (0.73-1.24)	1.00 (0.77-1.30)	1.01 (0.78-1.32)
5+ years after	0.67* (0.51-0.88)	0.86 (0.65-1.14)	0.89 (0.67-1.17)
**Procedural abortion**			
1 year before	1.00 (reference)	1.00 (reference)	1.00 (reference)
<1 year after	1.10 (0.92-1.31)	1.07 (0.90-1.28)	1.08 (0.90-1.29)
1-<2 years after	1.03 (0.86-1.24)	0.99 (0.82-1.19)	1.00 (0.83-1.20)
2-<5 years after	1.02 (0.88-1.19)	0.97 (0.93-1.13)	0.98 (0.84-1.15)
5+ years after	0.75** (0.65-0.87)	0.86 (0.74-1.01)	0.87 (0.74-1.02)
**Outcome: Any First Antianxiety Prescription**			
**Medication abortion**			
1 year before	1.00 (reference)	1.00 (reference)	1.00 (reference)
<1 year after	0.99 (0.61-1.59)	1.00 (0.62-1.62)	1.01 (0.62-1.63)
1-<2 years after	0.98 (0.59-1.60)	1.00 (0.61-1.65)	1.02 (0.62-1.67)
2-<5 years after	0.92 (0.61-1.39)	0.97 (0.64-1.47)	1.00 (0.66-1.51)
5+ years after	0.97 (0.65-1.44)	1.07 (0.71-1.60)	1.14 (0.76-1.71)
**Procedural abortion**			
1 year before	1.00 (reference)	1.00 (reference)	1.00 (reference)
<1 year after	1.19 (0.92-1.54)	1.20 (0.93-1.56)	1.22 (0.94-1.57)
1-<2 years after	0.76 (0.56-1.02)	0.78 (0.58-1.05)	0.79 (0.59-1.07)
2-<5 years after	1.07 (0.86-1.33)	1.12 (0.89-1.41)	1.15 (0.91-1.45)
5+ years after	0.81 (0.66-1.01)	0.93 (0.74-1.18)	0.98 (0.77-1.25)

© 2026 Steinberg JR et al. *JAMA Psychiatry*.

Notes. Relative to the year before an abortion, there are no increased risk after a medication or procedural abortion, respectively, in any first psychotropic medication redemption, any first antidepressant redemption, and any first antianxiety medication redemption.
[a]For each time period after the abortion, the reference is the year before the abortion within the method.
[b]Model includes abortion method (medication and procedural abortion) and cohort (calendar year).
[c]Model includes abortion method (medication and procedural abortion), cohort (calendar year), age and partner status at time of abortion, psychiatric diagnosis before one year before one's abortion, childbirth history and Charlson comorbidity index before one year before one's abortion, father presence during childhood, mother and father income at age 12, and mother and father psychotropic drug use before one year before one's abortion.
*$p < 0.05$, **$p < 0.002$ (the Bonferroni-corrected p-value).

© 2026 Steinberg JR et al. *JAMA Psychiatry*.

eTable 2. Incidence Rate Ratios (and 95% CI) of First Psychotropic Medication Prescription, Antidepressant Prescription, and Antianxiety Prescription at Each Time Relative to 1 Year Before the Abortion (n = 67,390) Where 2,500 Women With Both Medication and Procedural Codes Are Placed in the Medication Abortion Group

	**Unadjusted**	**Basic Model***	**Fully adjusted model****
**Outcome: Any First Psychotropic Medication Prescription**			
**Medication abortion**			
1 year before	1.00 (reference)	1.00 (reference)	1.00 (reference)
<1 year after	1.07 (0.997-1.16)	1.09* (1.02-1.18)	1.10* (1.02-1.18)
1-<2 years after	1.03 (0.95-1.11)	1.06 (0.98-1.14)	1.06 (0.98-1.15)
2-<5 years after	0.93* (0.87-0.99)	0.98 (0.92-1.05)	0.99 (0.92-1.05)
5+ years after	0.74** (0.69-0.79)	0.86** (0.80-0.92)	0.85** (0.79-0.91)
**Procedural abortion**			
1 year before	1.00 (reference)	1.00 (reference)	1.00 (reference)
<1 year after	1.10* (1.03-1.19)	1.08* (1.01-1.16)	1.09* (1.01-1.17)
1-<2 years after	1.01 (0.94-1.09)	0.98 (0.91-1.06)	0.99 (0.92-1.07)
2-<5 years after	0.98 (0.92-1.04)	0.95 (0.89-1.01)	0.96 (0.90-1.02)
5+ years after	0.77** (0.72-0.82)	0.83** (0.78-0.88)	0.83** (0.78-0.89)
**Outcome: First Antidepressant Prescription**			
**Medication abortion**			
1 year before	1.00 (reference)	1.00 (reference)	1.00 (reference)
<1 year after	1.03 (0.94-1.13)	1.06 (0.97-1.17)	1.07 (0.97-1.17)
1-<2 years after	1.01 (0.92-1.11)	1.06 (0.96-1.17)	1.06 (0.96-1.17)
2-<5 years after	0.87** (0.80-0.95)	0.96 (0.88-1.04)	0.96 (0.88-1.04)
5+ years after	0.68** (0.62-0.74)	0.87* (0.80-0.95)	0.86** (0.79-0.95)
**Procedural abortion**			
1 year before	1.00 (reference)	1.00 (reference)	1.00 (reference)
<1 year after	1.15* (1.05-1.26)	1.12* (1.02-1.23)	1.13* (1.03-1.23)
1-<2 years after	1.06 (0.96-1.17)	1.02 (0.93-1.12)	1.03 (0.94-1.13)
2-<5 years after	1.06 (0.98-1.14)	1.02 (0.94-1.10)	1.03 (0.95-1.12)
5+ years after	0.78** (0.72-0.84)	0.90* (0.83-0.98)	0.91* (0.84-0.99)
**Outcome: Any First Antianxiety Prescription**			
**Medication abortion**			
1 year before	1.00 (reference)	1.00 (reference)	1.00 (reference)
<1 year after	1.22* (1.06-1.40)	1.24* (1.07-1.42)	1.25** (1.08-1.44)
1-<2 years after	1.07 (0.92-1.24)	1.09 (0.94-1.27)	1.12 (0.96-1.30)
2-<5 years after	1.15* (1.01-1.30)	1.20* (1.06-1.36)	1.25** (1.10-1.41)
5+ years after	0.99 (0.87-1.12)	1.07 (0.94-1.22)	1.17* (1.02-1.33)
**Procedural abortion**			
1 year before	1.00 (reference)	1.00 (reference)	1.00 (reference)
<1 year after	1.11 (0.97-1.28)	1.12 (0.98-1.29)	1.14 (0.99-1.30)
1-<2 years after	0.96 (0.83-1.11)	0.97 (0.84-1.12)	0.99 (0.86-1.15)
2-<5 years after	0.96 (0.85-1.07)	0.99 (0.88-1.12)	1.03 (0.92-1.17)
5+ years after	0.87* (0.78-0.97)	0.99 (0.88-1.12)	1.08 (0.96-1.22)

© 2026 Steinberg JR et al. *JAMA Psychiatry*.

Notes. Relative to the year before an abortion, there was an increased risk in first antianxiety medication redemption in the year after and 2-5 years after a medication abortion, $p \leq 0.002$.
[a]For each time period after the abortion, the reference is the year before the abortion within the method
[b]Model includes abortion method (medication and procedural abortion) and cohort (calendar year)
[c]Model includes abortion method (medication and procedural abortion), cohort (calendar year), and age, gestational age, and partner status at time of abortion, psychiatric diagnosis before one year before one's abortion, childbirth history and Charlson comorbidity index before one year before one's abortion, father presence during childhood, mother and father income at age 12, and mother and father psychotropic drug use before one year before one's abortion.
*$p < 0.05$, **$p < 0.002$ (the Bonferroni-corrected p-value).

© 2026 Steinberg JR et al. *JAMA Psychiatry*.

eFigure 1. Incidence Rate of Any Psychotropic Medication Use in Year Before and Year After Abortion (a) for First Procedural Abortion by Gestational Age at Time of Abortion, and (b) for First Medication Abortion by Gestational Age at Time of Abortion

(a) First procedural abortion by gestational age at time of abortion

(a.1) < 8 completed weeks (<56 days) gestation

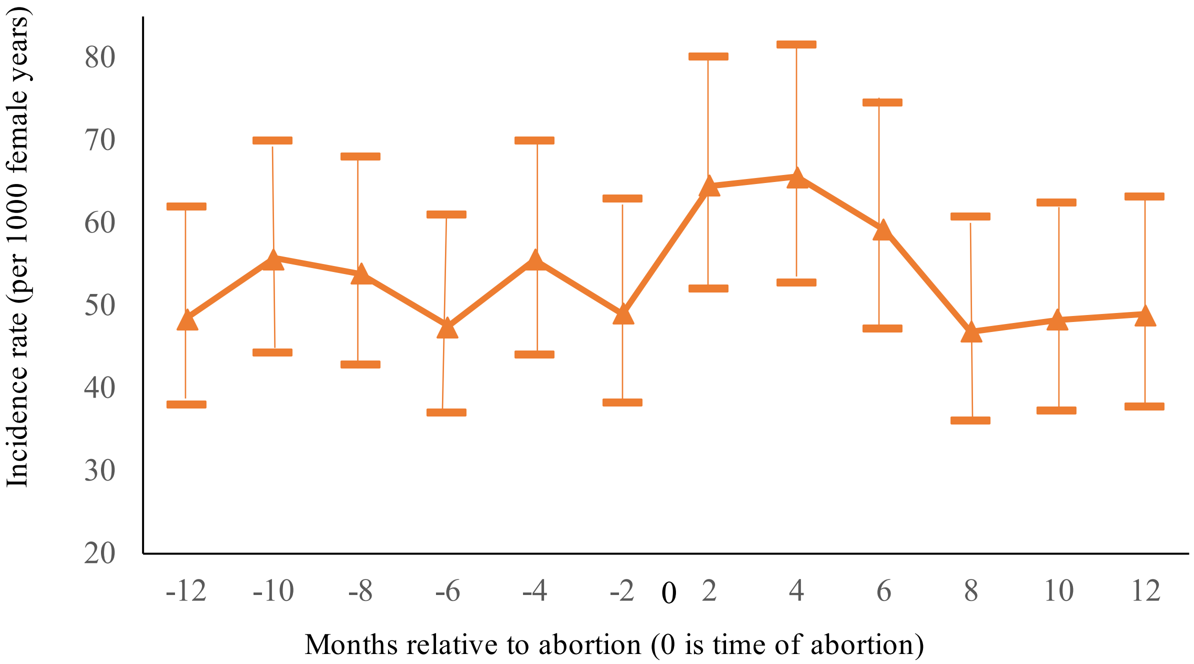

(a.2) 8 completed weeks (56 to 62 days) gestation

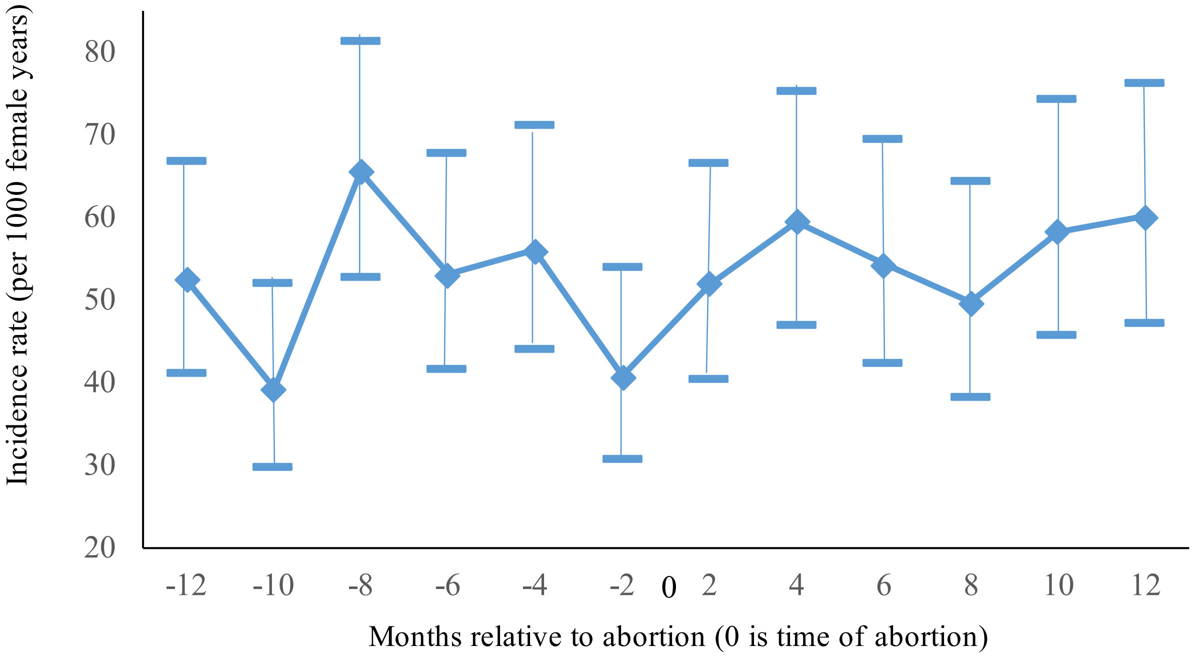

© 2026 Steinberg JR et al. *JAMA Psychiatry*.

(a.3) 9-11 complete weeks (63 days to 83 days) gestation

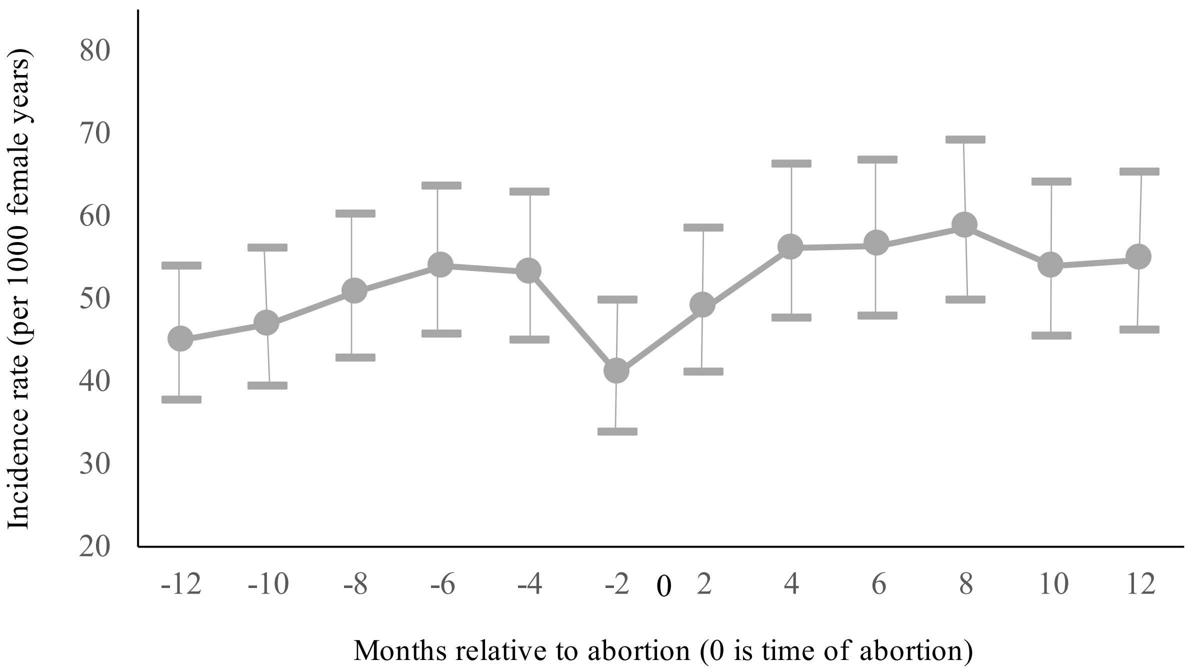

(b) First medication abortion by gestational age at time of abortion
(b.1) < 8 weeks (<56 days) gestation

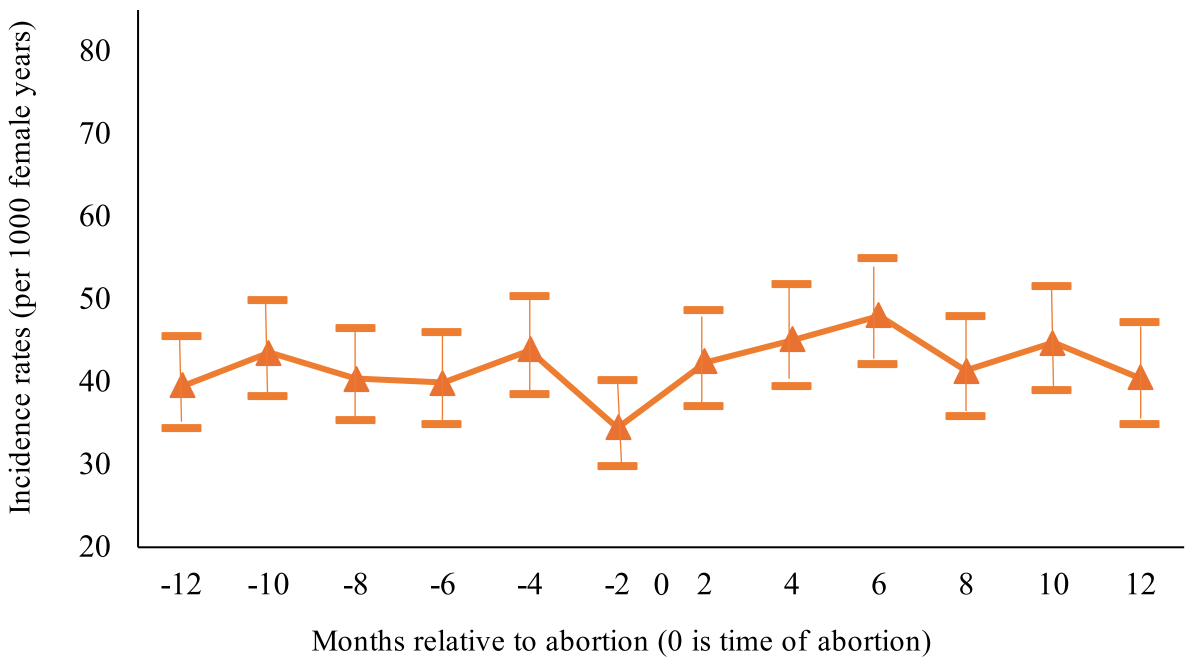

© 2026 Steinberg JR et al. *JAMA Psychiatry*.

(b.2) 8 weeks (56 to 62 days) gestation

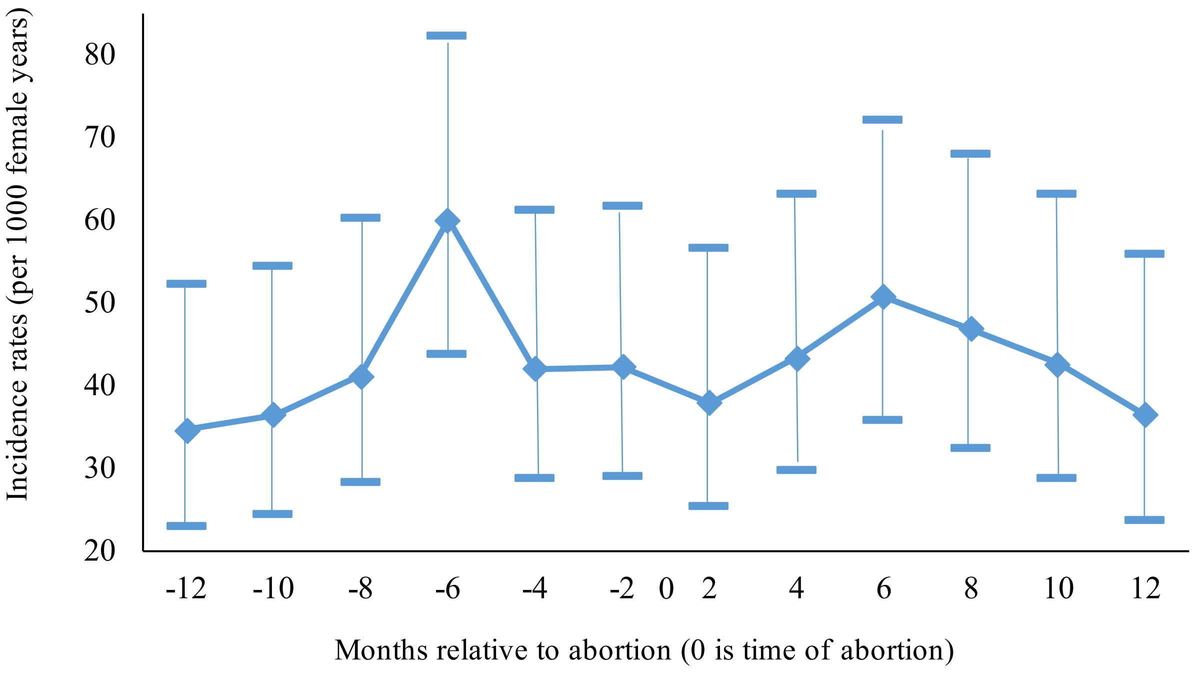

(b.3) 9 completed -11 completed weeks (63 days to 83 days) gestation

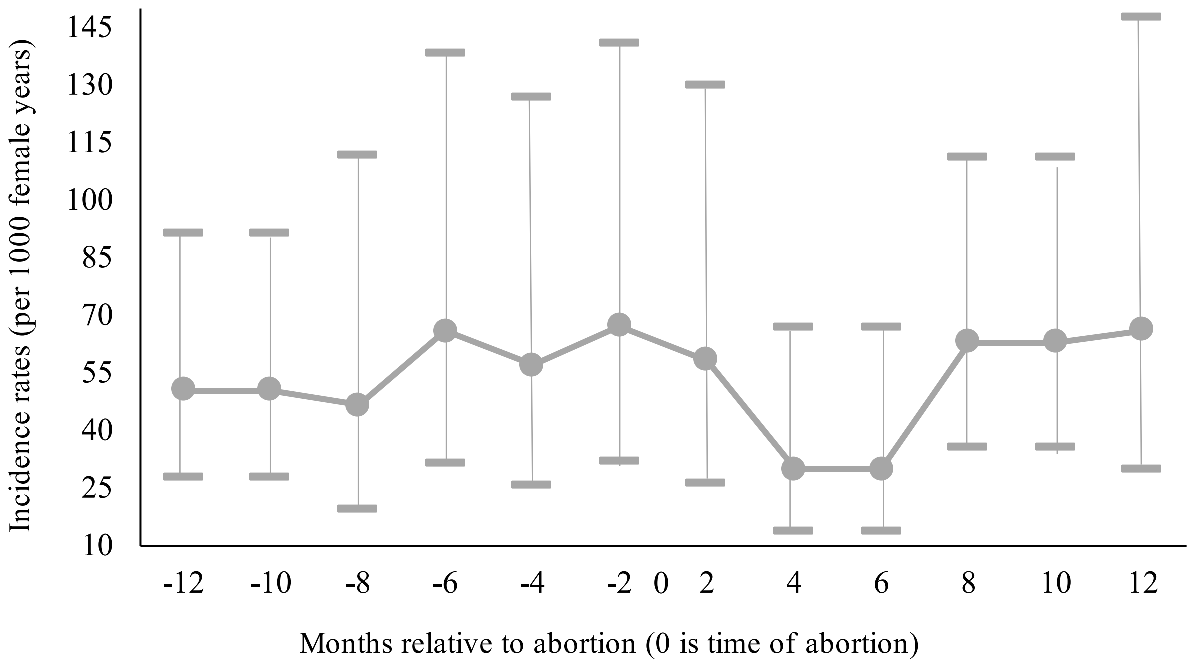

Notes: The period -12 to 0 refers to the period of 12 months before the abortion.

< 8 completed weeks (<56 days) | 8 completed weeks (56-62 days) | 9-11 completed weeks (63-83 days)

© 2026 Steinberg JR et al. *JAMA Psychiatry*.

eFigure 2. Incidence Rate Ratios of Any Psychotropic Medication Use in Year Before and Year After Abortion, Adjusted for Calendar Year for Females Having Had a (a) First Procedural Abortion by Gestational Age at Time of Abortion, and (b) First Medication Abortion by Gestational Age at Time of Abortion. Reference is incidence rates 11th and 12th months before the abortion.

(a) First procedural abortion by gestational age at time of abortion

(a.1) < 8 completed weeks (<56 days) gestation

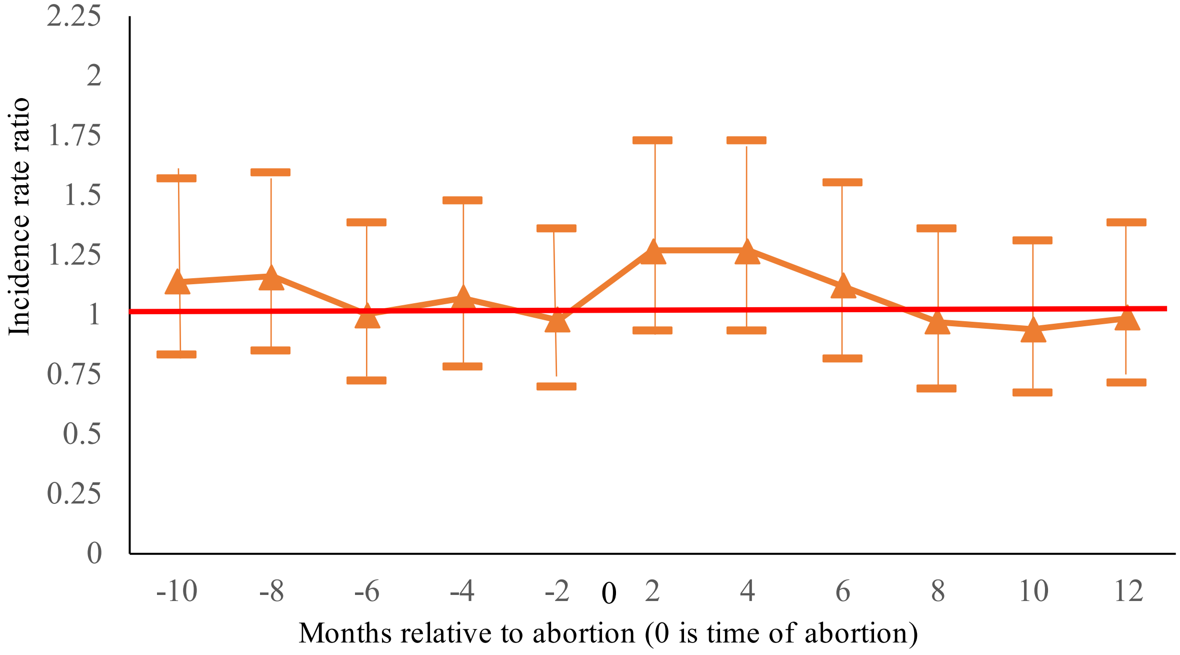

(a.2) 8 completed weeks (56 to 62 days) gestation

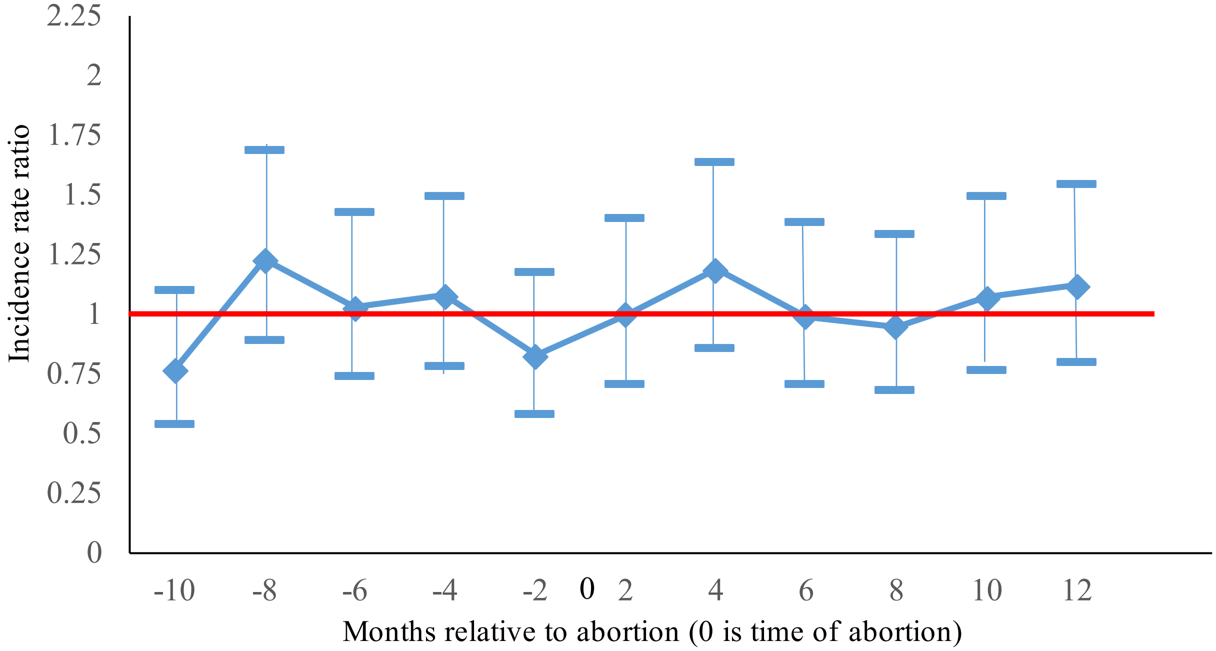

© 2026 Steinberg JR et al. *JAMA Psychiatry*.

(a.3) 9-11 completed weeks (63 to 83 days) gestation

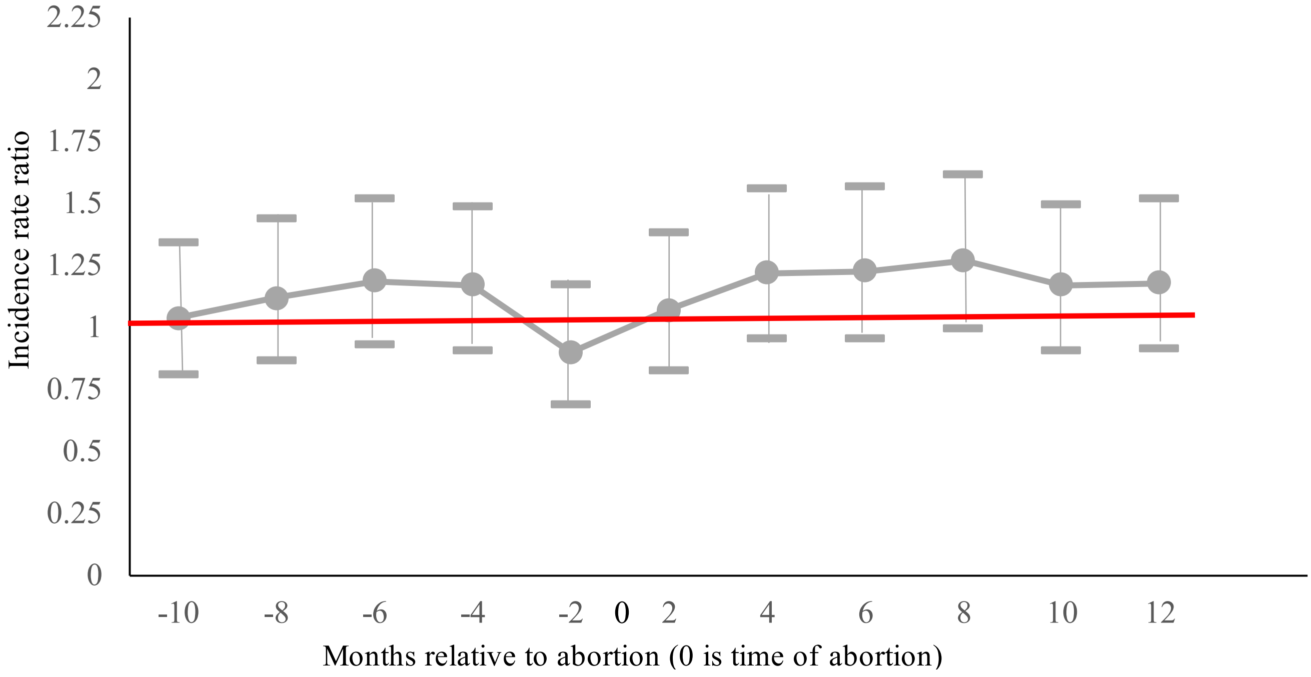

(b) First medication abortion by gestational age at time of abortion

(b.1) < 8 completed weeks (<56 days) gestation

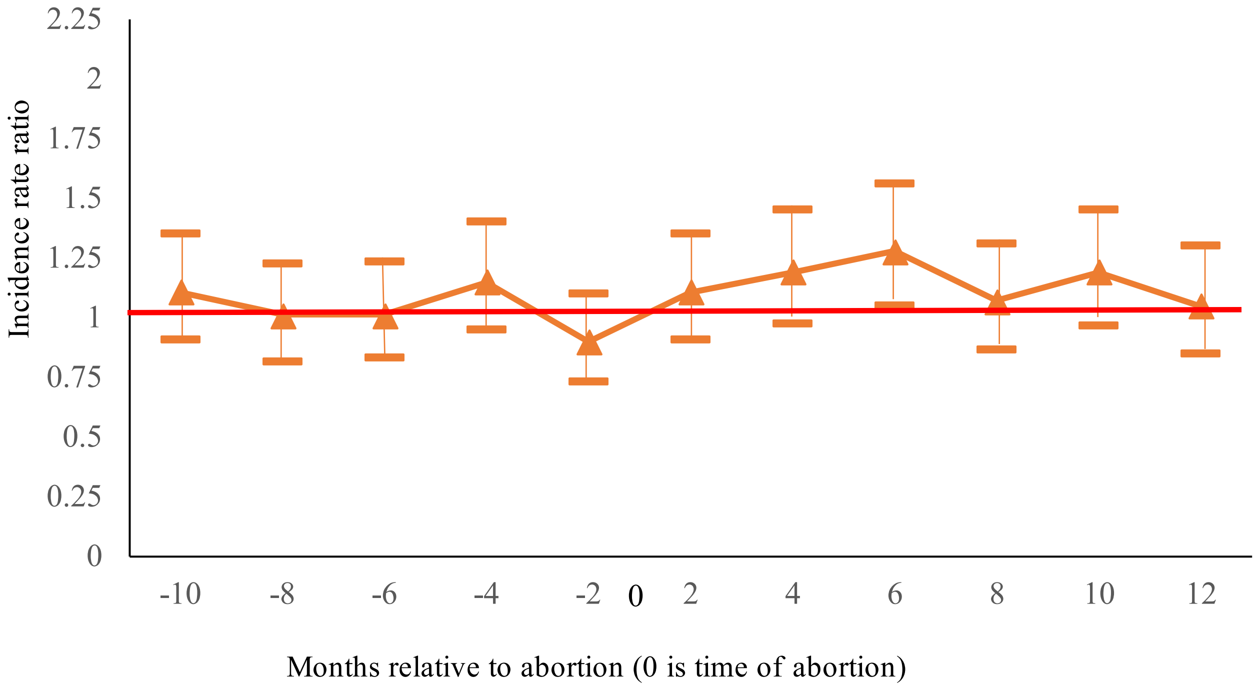

© 2026 Steinberg JR et al. *JAMA Psychiatry*.

(b.2) 8 completed weeks (56 to 62 days) gestation

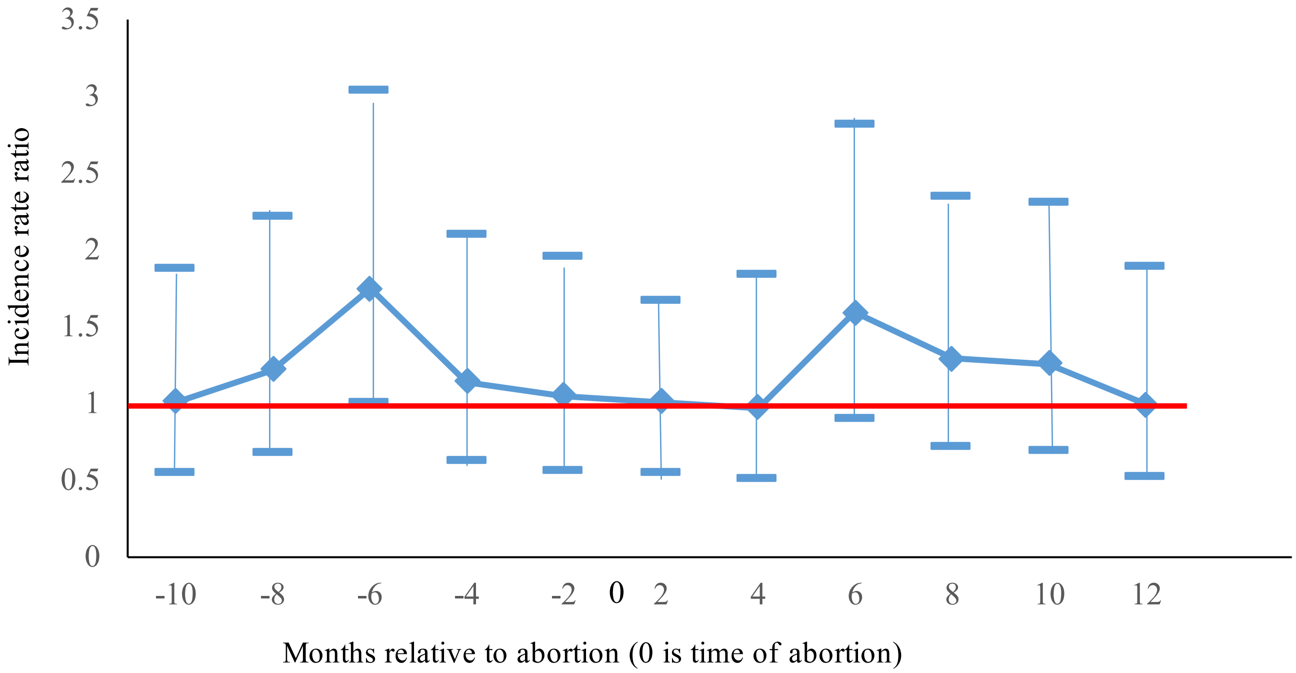

(b.3) 9-11 completed weeks (63 to 83 days) gestation

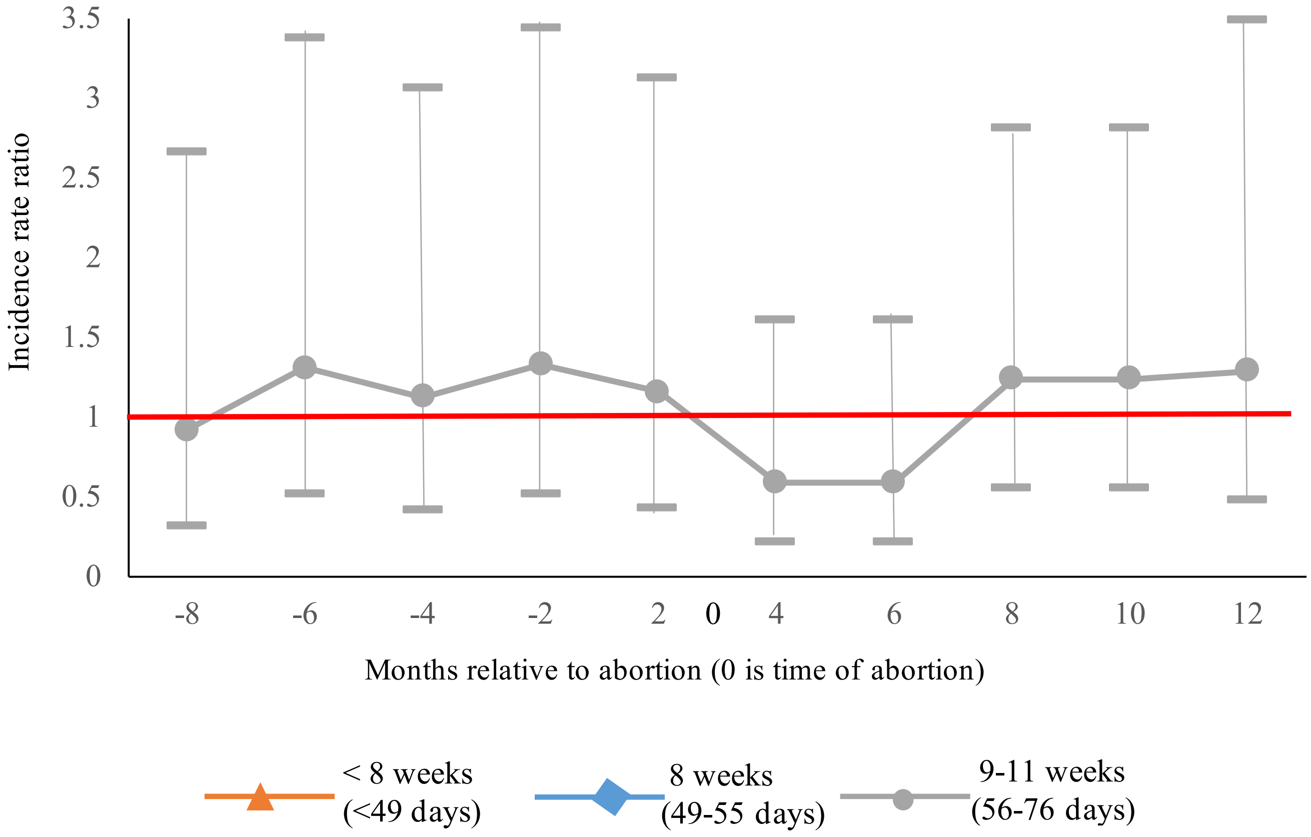

© 2026 Steinberg JR et al. *JAMA Psychiatry*.

Notes: The period -10 to 0 refers to the period of 10 months before the abortion. The solid horizontal line is the reference of 1.00, which is the 11th and 12th month before the abortion method within each gestational age group respectively with the exception of b.3. medication abortion at 9-11 completed weeks gestation. The reference for b.3. medication abortion at 9-11 completed weeks gestation is the 9th to 12th months before the abortion because there were not enough females with the outcome in the 11th and 12th month beforehand. The bars around each point are 95% confidence intervals of the estimate. Within each abortion method and gestational age group, there were only statistically significant higher rates from 4-6 months after a medication abortion at less than 8 completed weeks gestation, $p = 0.013$, and 5 to 6 months before a medication abortion at 8 completed weeks gestation, $p = 0.049$, at conventional p-values. These were no longer statistically significantly higher rates when using the Bonferroni-corrected p-value = 0.0045.

© 2026 Steinberg JR et al. *JAMA Psychiatry*.
