# Supplementary material for: Medication and Procedural Abortion and Risk of Psychotropic Medication Use
Source: JAMA Psychiatry. 2026 Jul 22:e253698. Online ahead of print. doi: 10.1001/jamapsychiatry.2025.3698 (PMC13392831; doi:10.1001/jamapsychiatry.2025.3698)
Supplement: Supplement 2. — Data Sharing Statement [file jamapsychiatry-e253698-s002.pdf]

Data Sharing Statement

Steinberg. Medication and Procedural Abortion and Risk of Psychotropic Medication Use. *JAMA Psychiatry*. Published July 22, 2026. doi:10.1001/jamapsychiatry.2025.3698

Data
Data available: No

Additional Information
Explanation for why data not available: Data are the Danish population registers and not made available to all per Danish rules and laws.
